# *E2F1*-mediated *KDM4A-AS1* up-regulation promotes EMT of hepatocellular carcinoma cells by recruiting *ILF3* to stabilize *AURKA* mRNA

**DOI:** 10.1038/s41417-023-00607-0

**Published:** 2023-03-27

**Authors:** Hao-Ming Shen, Di Zhang, Ping Xiao, Bin Qu, Yi-Fan Sun

**Affiliations:** 1 Hunan Key Laboratory of Oncotarget Gene, Hunan Cancer Hospital, The Affiliated Cancer Hospital of Xiangya School of Medicine, Central South University, Changsha, 410013 Hunan China; 2 Department of Clinical Laboratory, The Third Xiangya Hospital of Central South University, Changsha, 410013 Hunan China; 3 Department of Clinical Laboratory, The Eighth Affiliated Hospital of Guangxi Medical University, Guigang City People’s Hospital, Guigang, 537100 Guangxi China

**Keywords:** Cancer, Diseases

## Abstract

Hepatocellular carcinoma (HCC) is a gastrointestinal tumor with high clinical incidence. Long non-coding RNAs (lncRNAs) play vital roles in modulating the growth and epithelial-mesenchymal transition (EMT) of HCC. However, the underlying mechanism of lncRNA KDM4A antisense RNA 1 (KDM4A-AS1) in HCC remains elusive. In our study, the role of KDM4A-AS1 in HCC was systematically investigated. The levels of KDM4A-AS1, interleukin enhancer-binding factor 3 (ILF3), Aurora kinase A (AURKA), and E2F transcription factor 1 (E2F1) were determined by RT-qPCR or western blot. ChIP and dual luciferase reporter experiments were performed to detect the binding relationship between E2F1 and KDM4A-AS1 promoter sequence. RIP and RNA-pull down confirmed the interaction of ILF3 with KDM4A-AS1/AURKA. Cellular functions were analyzed by MTT, flow cytometry, wound healing and transwell assays. IHC was performed to detect Ki67 in vivo. We found that KDM4A-AS1 was increased in HCC tissues and cells. Elevated KDM4A-AS1 level was correlated to poor prognosis of HCC. Knockdown of KDM4A-AS1 inhibited the proliferation, migration, invasion and EMT of HCC cells. ILF3 bound to KDM4A-AS1 and AURKA. KDM4A-AS1 maintained the stability of AURKA mRNA by recruiting ILF3. E2F1 transcriptionally activated KDM4A-AS1. Overexpressed KDM4A-AS1 reversed the contribution of E2F1 depletion to AURKA expression and EMT in HCC cells. KDM4A-AS1 promoted tumor formation in vivo through the PI3K/AKT pathway. These results revealed that E2F1 transcriptionally activated KDM4A-AS1 to regulate HCC progression via the PI3K/AKT pathway. E2F1 and KDM4A-AS1 may serve as good prognostic targets for HCC treatment.

## Introduction

Hepatocellular carcinoma (HCC) accounts for 75–85% of primary liver cancers [1]. As one of the most malignancies worldwide, it ranks fifth in incidence and third in mortality [2]. Several major risk factors for HCC in recent years are alcohol consumption, smoking, obesity, and type 2 diabetes [3]. Chronic hepatitis B/C virus carriers are highly likely to progress to HCC [4]. In recent years, there have been various therapeutic approaches for HCC, including surgical resection, liver transplantation, local radiotherapy, and systemic chemotherapy [5]. However, the overall morbidity and mortality of HCC remain high, with aggressive growth behavior and a high recurrence rate, and most HCC patients often die of metastasis [6]. Thus, it is essential to identify the molecular mechanisms involved in HCC initiation and development.

Long non-coding RNAs (lncRNAs) are a new class of noncoding RNAs (>200 nucleotides) that act as important genetic modulators. LncRNAs are involved in cancer progression, including HCC. For example, lncRNA CDKN2BAS was associated with poor prognosis of HCC and promoted metastasis [7]. LncRNA PNUTS activated epithelial-mesenchymal transition (EMT) process in HCC [8]. What’s more, lncRNA CRNDE induced HCC growth and metastasis [9]. KDM4A antisense RNA 1 (KDM4A-AS1), a recently discovered lncRNA, acts as a tumor promoter. It was found that the inhibition of KDM4A-AS1 could reduce the viability, proliferation, migration and tumor growth of prostate cancer cells [10]. Recently, KDM4A-AS1 has been considered as one of the markers for measuring the overall survival rate of HCC [11]. However, there are few reports on the molecular mechanisms of KDM4A-AS1 in HCC.

Interleukin enhancer-binding factor 3 (ILF3) is an RNA-binding protein that can participate in regulating cell proliferation and angiogenesis in cancer cells [12]. It was reported that ILF3 promoted cell proliferation and transformation in various cancers [13]. Moreover, down-regulation of ILF3 suppressed cell proliferation in lung cancer [14]. Meanwhile, ILF3 can bind to lncRNAs to regulate downstream gene expression [15]. For example, lncRNA ILF3-AS1 promoted HCC progression through stabilizing ILF3 mRNA [16]. Nevertheless, the specific role of ILF3 in HCC remains to be further studied. The Starbase database predicted the presence of a binding site for KDM4A-AS1 with ILF3. Therefore, we speculated that KDM4A-AS1 might regulate the stability of downstream molecules by recruiting ILF3, thereby regulating the occurrence and development of HCC.

Aurora kinase A (AURKA) is a crucial member of the aurora kinase family and plays an important role during cell division [17]. Research has revealed that AURKA was critical in regulating tumor development and progression, including breast cancer [18], ovarian cancer [19] and prostate cancer [20]. Recently, it has been shown that AURKA was increased in HCC cells and tissues, and promoted HCC cell migration and invasion [21]. Meanwhile, AURKA gene could be used as a crucial predictor for HCC [22]. In addition, bioinformatics databases predicted that AURKA could be a bonding target of ILF3. Therefore, we speculated that ILF3 might promote the occurrence and development of HCC through stabilizing AURKA mRNA.

Herein, we explored the role of KDM4A-AS1 in HCC progression. We speculated that KDM4A-AS1 might promote EMT in HCC cells by recruiting ILF3 to stabilize AURKA mRNA. This research provides novel therapeutic targets for HCC treatment.

## Materials and methods

### Clinical samples

Tumor tissues and adjacent non-tumor tissues of 48 paired HCC cases were obtained from Hunan Cancer Hospital, The Affiliated Cancer Hospital of Xiangya School of Medicine, Central South University. All HCC patients did not receive any treatment before surgery. Tissues were stored at −80 °C. All procedures were approved by the Ethics Committee of Hunan Cancer Hospital. Signed informed consent was obtained from each patient.

### Cell culture and cell transfection

Human HCC cell lines (Hep 3B, HCCLM3, MHCC97-L, Huh-7) and human normal liver cell line (THLE-3) were obtained from the ATCC (Manassas, VA, USA) and Chinese Academy of Sciences (Cell Resource Center, Shanghai Institutes of Biological Sciences, Shanghai, China). Cells were grown in DMEM (Invitrogen, Carlsbad, CA, USA) supplemented with 10% fetal bovine serum (Invitrogen) at 37 °C with 5% CO_2_. The short hairpin RNAs (sh-RNAs) targeting KDM4A-AS1 (sh-KDM4A-AS1) and ILF3 (sh-ILF3) were designed and synthesized by GenePharma (Shanghai, China). The full-length cDNA of AURKA was synthesized by GenePharma and cloned into pcDNA 3.1 vector. Lentiviral vector expressing sh-E2F1 or OE-KDM4A-AS1 was constructed by GenePharma. Besides, their negative control groups (sh-NC, pcDNA 3.1, OE-NC) were also bought from GenePharma. Cells were infected with lentiviral particles in the presence of 10 μg/ml polybrene and selected using puromycin for one week. Lipofectamine 3000 reagent (Invitrogen) was utilized to perform cell transfection based on manufacturer’s instruction. Cells in the Control group grew normally without any treatment.

### Quantitative real-time PCR (RT-qPCR)

Total RNA was isolated using Trizol reagent (Invitrogen). cDNA was synthesized with PrimeScript RT Reagent Kit (Takara, Japan). Gene expression was analyzed using the SYBR Green PCR Kit (Sigma, MO, USA). GAPDH was conducted for endogenous control. 2^−∆∆Ct^ method was utilized to perform data analyses. Following primers were used:

KDM4A-AS1 F: 5′-TTGCCTGGATGGCTGAGAATC-3′, R: 5′-TTCCTTTCACCCTCCTTCCTTC-3′; ILF3 F: 5′-ACAGCAACGGGAAGATATCAC-3′, R: 5′-CCACTGGGTTTTCATTCTTTGG-3′; AURKA F: 5′-CTGAGGAGGAACTGGCATCAA-3′, R: 5′-ATTAGGTAGACTCTGGTAGCATCAT-3′; E2F1 F: 5′-ACGCTATGAGACCTCACTGAA-3′, R: 5′-TCCTGGGTCAACCCCTCAAG-3′; GAPDH F: 5′-GGTGTGAACCATGAGAAGTATGA-3′, R: 5′-GAGTCCTTCCACGATACCAAAG-3′. For AURKA mRNA stability detection, cells (2 × 10^4^/well) were plated in 12-well plates overnight. Then cells were exposed to 5 μg/ml actinomycin D (A9415, Sigma) for 0, 3, 6, 9, 12 h respectively. Cells were then collected and total RNA was isolated. RT-qPCR was used to determine the expression of AURKA.

### RNA in situ hybridization (FISH)

The FISH kit (RiboBio, Guangzhou, China) was applied for detecting KDM4A-AS1’s subcellular localization according to previous description [23]. In brief, HCC cells were plated on glass coverslips into 24-well plates (1 × 10^5^/well) for 24 h. 4% paraformaldehyde was used to fix cells cultured on coverglass. After fixation, cells were subject to 5-min permeabilization with 0.5% Triton X-100. Next, cells were incubated with prehybridization solution and hybridized with hybridization solution, and then incubated with Cy3-labeled KDM4A-AS1 probe overnight. Then we washed the glasses with washing buffer and DAPI was utilized to stain nuclei. The fluorescence microscope (Olympus, Tokyo, Japan) was adopted for observing cells and acquiring images.

### Western blot

RIPA buffer was used to extract total protein. Proteins were quantified using a BCA protein determination Kit (KeyGEN Biotech, Nanjing, China). 30 μg protein was separated by 10% SDS-PAGE and transferred to PVDF membranes. The membranes were blocked in 5% non-fat milk for 1 h. The incubation of blots was conducted with antibodies at 4 °C overnight: Bax (ab32503, 1:1000, Abcam, Cambridge, MA, USA), Bcl-2 (ab117115, 1:1000, Abcam), ILF3 (ab225626, 1:1000, Abcam), AURKA (ab108353, 1:1000, Abcam), E2F1 (ab4070, 1:500, Abcam), E-cadherin (ab231303, 1:1000, Abcam), Vimentin (ab92547, 1:1000, Abcam), PI3K (#3811, 1:1000, CST, Danvers, MA, USA), phosphorylated PI3K (p-PI3K, #4228, 1:1,000, CST), AKT (#9272, 1:1,000, CST), phosphorylated AKT (p-AKT, #9271, 1:1000, CST) or GAPDH (ab8245, ab9485, 1:5000, Abcam) and then with HRP-conjugated second antibody (#7074, 1:1000, CST). Protein bands were detected with ECL Plus reagent (Pharmacia, Piscataway, USA), and visualized using a Gel Imaging System. Bands were then quantified using ImageJ software (National Institutes of Health). The expression of GAPDH was used for data normalization.

### Apoptosis analysis

HCC cell apoptosis was analyzed using flow cytometry (FCM). In brief, after rinsing twice by PBS, cells were plated in six-well plates (1 × 10^6^/well) and subject to 15-min incubation using Annexin V-FITC (5 μL) as well as propidium iodide (PI, 5 μL, KeyGEN Biotech) in succession under ambient temperature in dark, followed by FCM evaluation (BD Biosciences, San Jose, CA, USA) together with the analysis with FlowJo (Version 7.6.5, TreeStar, Ashland, OR, USA).

### MTT assay

HCC cells (2 × 10^3^/well) were inoculated in 96-well plates for 24 h. 20 μL (5 mg/ml) of Thiazolyl blue (MTT) reagent (Promega, Madison, WI, USA) was added to each well. After 4 h incubation, 100 μL of dimethyl sulfoxide (DMSO) was added to each well and optical density (OD) was measured by the enzyme-link meter at 490 nm. The viability index was calculated as the (experimental OD value-blank OD value)/(control OD value-blank OD value) × 100%.

### Wound-healing assay

HCC cell migration was detected by a wound-healing assay. Transfected cells (2 × 10^5^) were plated on 6-well plates to grow to 80% confluence. After scratching with a sterile pipette tip, cells were incubated under standard conditions. The migrated cells were observed, and the distance was determined at 0 h and 24 h.

### Invasion assay

Transwell assay was carried out with the purpose of assessing cell invasion. To be specific, Transwell chamber (Corning, NY, USA) was precoated with Matrigel. Thereafter, cell suspension (1 × 10^5^/well) was prepared with serum-free medium (200 μL) within the top chamber. The lower chamber was added with DMEM supplemented with 10% FBS for 24 h incubation. Thereafter, 4% formaldehyde (PFA) was utilized to fix invading cells on the bottom chamber surface, followed by 0.1% crystal violet staining. Later, five randomly selected fields were counted with the microscope (Olympus).

### Dual-luciferase reporter assay

E2F1’s binding sites in KDM4A-AS1 or mutant sequences were cloned into pGL3-basic vector (Promega). After inoculation onto 24-well plates (1 × 10^5^/well), cells were subject to co-transfection using pGL3-KDM4A-AS1 promoter, sh-E2F1, or sh-NC. Cell collection was conducted after 48-h incubation, and the luciferase reporter assay system (Promega) was utilized to identify luciferase activities in line with specific instructions.

### Chromatin immunoprecipitation (ChIP) assay

HCC cells were plated into a 24-well plate at 1 × 10^5^/well. After 24 h, cells were collected and ChIP assay was performed using SimpleChIP® Enzymatic Chromatin IP Kit (CST). HCC cells (5 × 10^6^) were cross-linked with 37% formaldehyde. Chromatin fragments were generated by the sonication of cell lysates. DNA-protein complexes were immunoprecipitated using E2F1 antibody and IgG antibody. The precipitated chromatin DNA was analyzed using qPCR with KDM4A-AS1 primers.

### RNA immunoprecipitation (RIP)

Magna RIP Quad RNA-Binding Protein Immunoprecipitation Kit (Sigma) was utilized for RIP assay. To be specific, HCC cells were collected and lysed with RIP lysis buffer. Samples were centrifuged for 15 min at 4 °C. Supernatant was added to either IgG- or anti-ILF3 antibody-bound magnetic beads and incubated overnight under 4 °C. RNA was immunoprecipitated via magnetic beads and rinsed by lysis buffer thrice, the immunoprecipitate complex was collected and the immunoprecipitated RNA was analyzed with RT-qPCR.

### RNA pull-down

Biotin-labeled AURKA and scramble control, KDM4A-AS1 sense, and antisense control sequences were transfected into HCC cells. After 48 h, the cells were cross-linked with 1% formaldehyde for 10 min, then 0.125 M glycine was used to stop the reaction. Cells were then lysed in lysis buffer containing complete protease inhibitor and RNase inhibitor and incubated with streptavidin-conjugated agarose beads (Invitrogen) for 3 h at 4 °C. Afterward, the beads were washed with lysis buffer three times. The eluted proteins were determined with western blot using ILF3 antibody.

### Nude mice tumorigenesis

Male BALB/c nude mice (4 weeks old, Shanghai laboratory Animal Center of Chinese Academy of Sciences, Shanghai, China) were used and housed in a controlled environment (12 h light–dark cycle, 25 °C, and 60-70% humidity). Mice were randomly divided into four groups (*n* = 5 per group): Control, sh-NC + OE-NC, sh-E2F1 + OE-NC, sh-E2F1 + OE-KDM4A-AS1. HCC cells (Hep3B) with stable expression of sh-E2F1, OE-KDM4A-AS1, or negative controls were used. These stable cells were trypsinized and suspended in PBS. A total volume of 0.1 mL of PBS containing 1 × 10^6^ HCC cells was injected subcutaneously into the axilla of the right forelimb of mice (*n* = 5 mice/group). Mice in the Control group were treated with 0.1 mL of PBS. The tumor nodules were examined every 5 days. Tumors were surgically removed and weighed after 25 days. Tumor volumes were determined by the formula: *V* = (Width^2^ × Length)/2. All animal experiment protocols were permitted by the Animal Ethics Committee of Hunan Cancer Hospital.

### Immunohistochemistry (IHC)

Formalin-fixed tumor samples were paraffin-embedded and sectioned. After deparaffinization and rehydration, antigen retrieval was performed using 10 mmol/l citric acid buffer (pH 6.0) at 100 °C for 15 min. Next, sections were subjected to anti-Ki-67 antibody (1:200, ab15580, Abcam) at 4 °C overnight. After rinsing in PBS, samples were incubated with a secondary antibody for 1 h. Reactivity was developed in diaminobenzidine (DAB) (Beyotime, Shanghai, China). Slides were observed under light microscopy.

### Statistical analysis

SPSS 22.0 (IBM, Armonk, NY, USA) was used to perform statistical analysis. All experiments were conducted in triplicate. Data are presented as means ± SD. Differences between two groups were measured by a paired two-tailed *t* test. Statistical differences of more than two groups were evaluated using One-way ANOVA analysis. A *P* value <0.05 was considered statistically significant.

## Results

### Knockdown of KDM4A-AS1 inhibited EMT of HCC cells

**Fig. 1:**
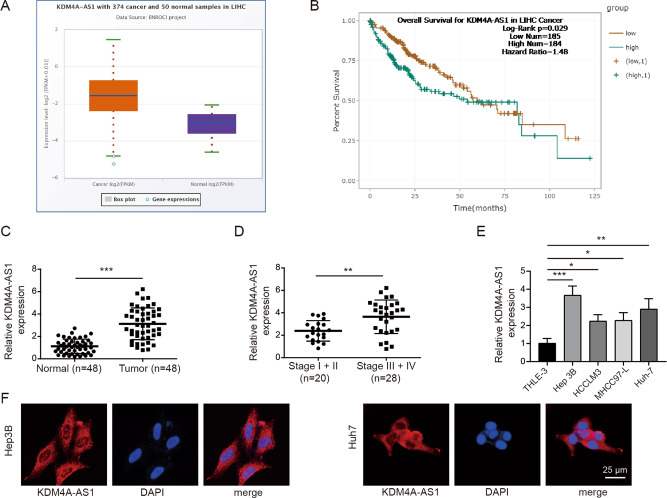
KDM4A-AS1 was up-regulated in HCC. **A** Starbase database predicted KDM4A-AS1 level in HCC tissues and normal adjacent tissues. **B** Kaplan–Meier analysis was used to detect the survival rate of HCC patients. **C** RT-qPCR was performed to examine KDM4A-AS1 level in HCC tissues (*n* = 48) and adjacent normal tissues (*n* = 48). **D** RT-PCR detected the level of KDM4A-AS1 in clinical phase I + II and III + IV tissues. **E** RT-qPCR analysis of KDM4A-AS1 level in human normal liver cells (THLE-3) and HCC cell lines. **F** FISH assay was utilized to test KDM4A-AS1 location in HCC cells. Results were expressed as means ± SD for at least triplicate experiments. *P < 0.05, **P < 0.01, ***P < 0.001.

**Fig. 2:**
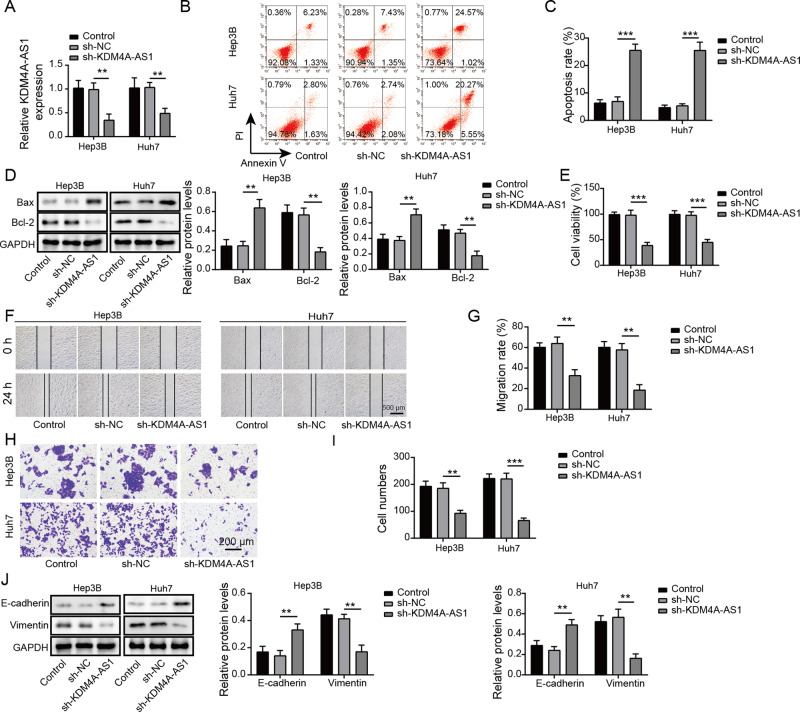
Knockdown of KDM4A-AS1 inhibited EMT of HCC cells. **A** Transfection efficiency was verified by RT-qPCR. **B**, **C** Flow cytometry was applied for cell apoptosis analysis. **D** Western blot was used to examine Bax and Bcl-2 expression. **E** MTT assay was conducted to determine cell viability. **F**, **G** Wound healing experiment examined cell migration. **H**, **I** Transwell analysis of cell invasion ability. **J** Western blot was performed to detect EMT-related proteins. Results were expressed as means ± SD for at least triplicate experiments. **P < 0.01, ***P < 0.001.

According to the Starbase database analysis, KDM4A-AS1 was increased in HCC tissues compared with normal adjacent tissues (Fig. 1A). Survival analysis identified that high level of KDM4A-AS1 was related to poor prognosis of HCC patients (Fig. 1B). Moreover, RT-qPCR analysis detected that KDM4A-AS1 level in HCC tissues was remarkably increased compared to normal adjacent tissues (Fig. 1C). The level of KDM4A-AS1 in HCC tumor tissues of stage III + IV patients was significantly higher than that in stage I + II, implying that expression level of KDM4A-AS1 was closely related to the severity of tumor (Fig. 1D). Furthermore, compared to control THLE-3 cells, KDM4A-AS1 expression in different HCC cell lines was generally increased (Fig. 1E). Hep 3B and Huh-7 cells with the most significant differences were chosen for the subsequent experiments. FISH detection found that KDM4A-AS1 was distributed in both nucleus and cytoplasm of HCC cells, and was mainly in the cytoplasm (Fig. 1F). Posteriorly, we further explored whether KDM4A-AS1 could influence HCC progression. KDM4A-AS1 was decreased in HCC cells when transfected with sh-KDM4A-AS1 compared that in sh-NC group (Fig. 2A). Moreover, the apoptosis rate of HCC cells increased markedly with KDM4A-AS1 silencing (Fig. 2B, C). Meanwhile, KDM4A-AS1 knockdown greatly increased Bax expression but decreased Bcl-2 expression (Fig. 2D). Additionally, KDM4A-AS1 depletion obviously inhibited HCC cell proliferation, migration and invasion (Fig. 2E–I). What’s more, we determined EMT-related markers (E-cadherin, Vimentin) by western blot and found that compared with sh-NC group, knockdown of KDM4A-AS1 obviously increased E-cadherin expression, but greatly decreased Vimentin expression (Fig. 2J). The above data suggested that KDM4A-AS1 regulated HCC cell growth and EMT.

### KDM4A-AS1 recruited ILF3 to maintain the stability of AURKA mRNA in HCC cells

**Fig. 3:**
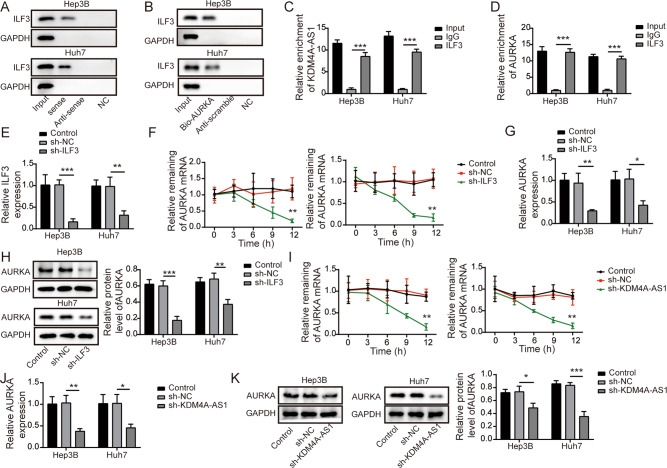
KDM4A-AS1 recruited ILF3 to maintain the stability of AURKA mRNA in HCC cells. **A** RNA pull-down verified KDM4A-AS1 could directly bind to ILF3. **B** RNA pull-down verified ILF3 specifically bonded to AURKA. **C** RIP confirmed the targeting relationship between KDM4A-AS1 and ILF3. **D** RIP assay confirmed AURKA as a target of ILF3. **E** The expression of ILF3 was tested by RT-qPCR. **F** Cells were exposed to 5 μg/ml actinomycin D for 0, 3, 6, 9, 12 h, respectively. RT-qPCR analysis of the mRNA stability of AURKA. **G**, **H** AURKA level was tested by RT-qPCR and Western blot after knocking down ILF3. **I** Detection of AURKA mRNA stability. **J**, **K** RT-qPCR and Western blot were used to detect AURKA level after knocking down KDM4A-AS1. Data were shown as means ± SD for three independent experiments. *P < 0.05, **P < 0.01, ***P < 0.001.

We then explored the molecular mechanism by which KDM4A-AS1 regulated EMT of HCC cells. RNA-pull down assay indicated that KDM4A-AS1 sense probe pulled down ILF3 (Fig. 3A). In addition, ILF3 from HCC lysates was also enriched by biotin-labeled AURKA (Fig. 3B). Furthermore, RIP assay verified that compared with the anti-IgG groups, both KDM4A-AS1 and AURKA were enriched in anti-ILF3 immunoprecipitated RNA (Fig. 3C, D). Then, compared to sh-NC group, ILF3 was successfully knocked down by sh-ILF3 (Fig. 3E). It could be seen in Fig. 3F that knockdown of ILF3 dramatically down-regulated the stability of AURKA mRNA in actinomycin D (5 μg/ml) treated cells. Additionally, ILF3 inhibition decreased AURKA expression (Fig. 3G, H). Furthermore, the stability of AURKA mRNA was also reduced after KDM4A-AS1 silencing (Fig. 3I). Meanwhile, both mRNA and protein levels of AURKA were markedly decreased by KDM4A-AS1 knockdown (Fig. 3J, K). The above results indicated that KDM4A-AS1 maintained the stability of AURKA mRNA by binding to ILF3.

### ILF3 knockdown inhibited EMT of HCC cells through inhibiting AURKA

**Fig. 4:**
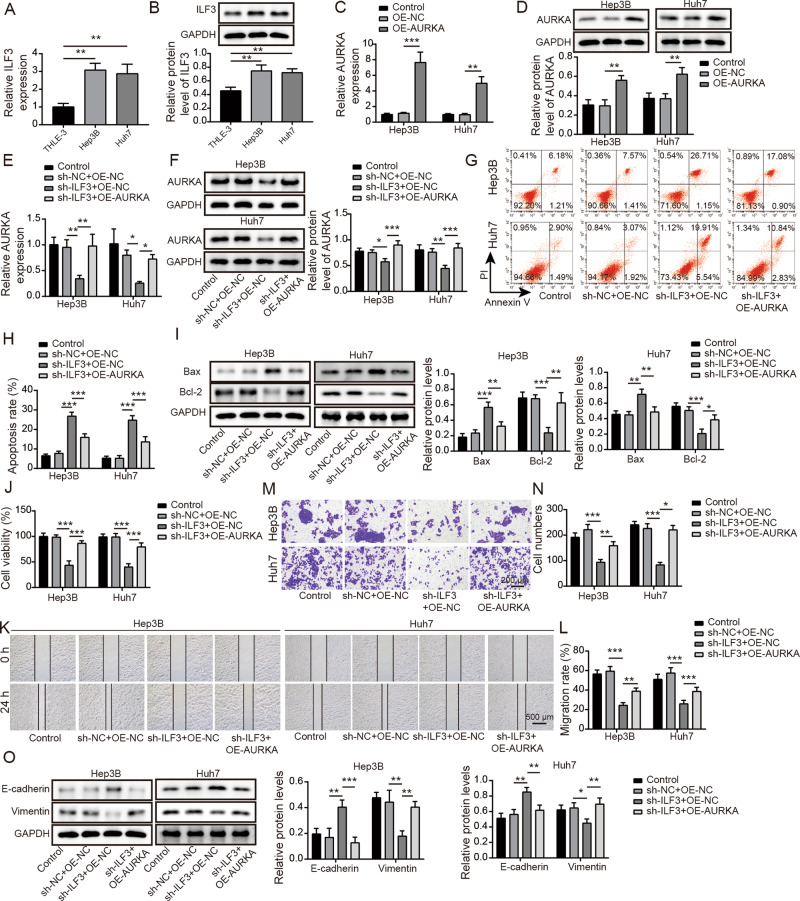
ILF3 knockdown inhibited EMT of HCC cells through inhibiting AURKA. **A**, **B** RT-qPCR and western blot were utilized to assess ILF3 level in THLE-3, Hep 3B, and Huh-7 cells. **C**, **D** The transfection efficiency of pcDNA 3.1 AURKA was verified by RT-qPCR and western blot. **E**, **F** RT-qPCR and western blot were used to detect AURKA expression in HCC cells with sh-ILF3 and pcDNA 3.1 AURKA. **G**, **H** Cell apoptosis was tested by flow cytometry. **I** Western blot was conducted to examine Bax and Bcl-2 expression. **J** MTT assay was performed to determine cell viability. **K**, **L** Wound healing experiments checked cell migration. **M**, **N** Transwell analysis of cell invasion ability. **O** Western blot detection of EMT-related proteins. Results are expressed as mean ± SD for at least triplicate experiments. *P < 0.05, **P < 0.01, ***P < 0.001.

We further found that ILF3 expression was remarkably increased in HCC cells compared with normal liver cells (Fig. 4A, B). Therefore, we detected the regulatory effect of ILF3 on AURKA in HCC by overexpressing AURKA and knocking down ILF3. Firstly, compared to pcDNA 3.1 vector group, the pcDNA 3.1 AURKA greatly up-regutated AURKA mRNA and protein level in HCC cells (Fig. 4C, D). Moreover, compared to negative control group, sh-ILF3 treatment down-regulated the level of AURKA, while overexpression of AURKA reversed the inhibitory effect of ILF3 knockdown (Fig. 4E, F). Furthermore, compared with negative control group, depletion of ILF3 promoted cell apoptosis, up-regulated Bax expression and down-regulated Bcl-2 protein level, while these effects were reversed after co-transfection with pcDNA 3.1-AURKA (Fig. 4G–I). Additionally, depression of ILF3 inhibited HCC cell proliferation, migration and invasion. However, these effects were overturned by AURKA overexpression (Fig. 4J–N). Meanwhile, ILF3 downregulation-mediated decrease in Vimentin expression and increase in E-cadherin were abrogated by AURKA upregulation (Fig. 4O). These results implied that overexpression of AURKA reversed the effect of ILF3 knockdown on HCC cell EMT.

### E2F1 facilitated KDM4A-AS1 transcription in HCC cells

**Fig. 5:**
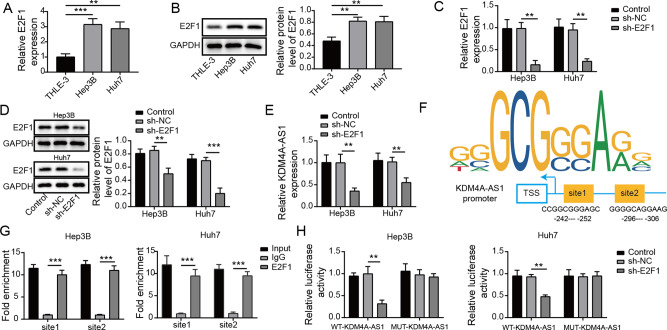
E2F1 facilitated KDM4A-AS1 transcription in HCC cells. **A**, **B** RT-qPCR and western blot were used to assess E2F1 levels in THLE-3, Hep 3B, and Huh-7 cells. **C**, **D** The transfection efficiency of sh-E2F1 was verified by RT-qPCR and western blot. **E** RT-qPCR was performed to assess KDM4A-AS1 level in HCC cells with sh-E2F1. **F** The binding site of E2F1 in the KDM4A-AS1 promoter was predicted by JASPER. **G**, **H** ChIP and luciferase reporter assays detected the binding relationship between E2F1 and KDM4A-AS1 promoter sequence. Data were shown as means ± SD for three independent experiments. *P < 0.05, **P < 0.01, ***P < 0.001.

For examining the mechanism of KDM4A-AS1 on HCC EMT, we found that E2F1 expression in HCC cells was obviously increased compared to normal liver cells (Fig. 5A, B). Next, compared with sh-NC group, E2F1 depletion significantly downregulated E2F1 and KDM4A-AS1 expression in HCC cells (Fig. 5C–E). We further predicted the binding sites of E2F1 in the KDM4A-AS1 promoter region using JASPAR (Fig. 5F). Furthermore, E2F1-binding DNA fragments were immunoprecipitated using ChIP analysis. The PCR product was amplified by the paired primers of KDM4A-AS1 promoter in the immunoprecipitate captured by E2F1 antibody, implying the combination of E2F1 and KDM4A-AS1 promoter region (Fig. 5G). Meanwhile, dual luciferase reporter analysis implied that with E2F1 knockdown, the binding of E2F1 to wild KDM4A-AS1 was reduced compared to sh-NC group, but the binding to mutant KDM4A-AS1 remained unchanged (Fig. 5H). Taken together, these findings revealed that E2F1 bound to KDM4A-AS1 promoter region and increased KDM4A-AS1 expression in HCC cells.

### E2F1 knockdown inhibited HCC cell EMT through KDM4A-AS1

**Fig. 6:**
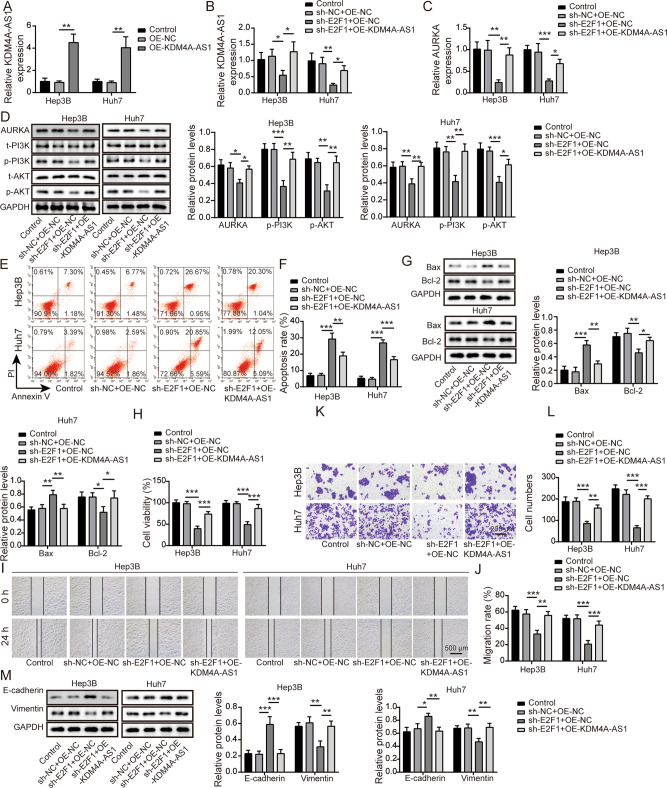
E2F1 knockdown inhibited HCC EMT through KDM4A-AS1. **A** The transfection efficiency of OE-KDM4A-AS1 was verified using RT-qPCR. **B** RT-qPCR was used to assess KDM4A-AS1 level in HCC cells with sh-E2F1 and OE-KDM4A-AS1. **C** RT-qPCR was utilized to detect AURKA expression. **D** Western blot was performed to detect AURKA and PI3K/AKT pathway protein levels. **E**, **F** Flow cytometry was employed to measure cell apoptosis. **G** Western blot analysis of Bax and Bcl-2 expression. **H** MTT assay was used to determine cell viability. **I**, **J** Wound healing experiment tested cell migration. **K**, **L** Transwell analysis of cell invasion ability. **M** Western blot was conducted to detect EMT-related proteins. Results were expressed as means ± SD for at least triplicate experiments. *P < 0.05, **P < 0.01, ***P < 0.001.

To better understand the correlation between E2F1 and KDM4A-AS1 in HCC, we overexpressed KDM4A-AS1 in Hep 3B and Huh-7 cells. As seen in Fig. 6A, OE-NC had no effect on KDM4A-AS1 expression, but OE-KDM4A-AS1 significantly increased KDM4A-AS level compared to OE-NC. We further revealed that E2F1 repression inhibited KDM4A-AS1 and AURKA expression compared with negative control group, while this inhibitory effect was overturned by co-transfection of OE-KDM4A-AS1 (Fig. 6B, C). Additionally, compared to negative control group, AURKA, p-PI3K and p-AKT expressions in HCC cells with sh-E2F1 were decreased, but OE-KDM4A-AS1 could restore these expression levels obviously (Fig. 6D). Moreover, the promotion effect of sh-E2F1 on cell apoptosis was reversed by co-transfection of OE-KDM4A-AS1 (Fig. 6E–G). In addition, compared with negative control group, cell proliferation, migration and invasion were remarkably suppressed by sh-E2F1, whereas OE-KDM4A-AS1 overexpression overturned these effects (Fig. 6H–L). Meanwhile, sh-E2F1 increased E-cadherin and deceased Vimentin levels compared to negative control group. When OE-KDM4A-AS1 was co-transfected, opposite results were observed (Fig. 6M). Therefore, overexpressed KDM4A-AS1 reversed the effects of E2F1 silencing on EMT of HCC cells.

### KDM4A-AS1 medicated by E2F1 promoted tumor formation in vivo via activating PI3K/AKT signaling

**Fig. 7:**
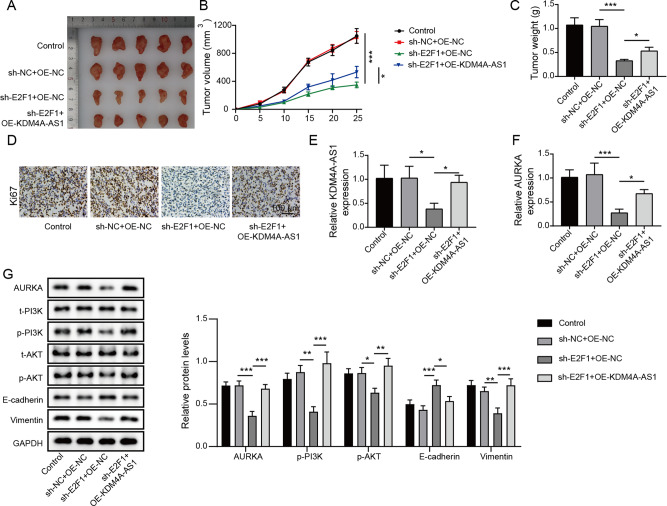
KDM4A-AS1 medicated by E2F1 promoted tumor formation in vivo via activating PI3K/AKT signaling. BALB/C male nude mice were injected subcutaneously with HCC cells transfected with stable sh-E2F1 and OE-KDM4A-AS1. **A** The images of the xenograft tumors. **B** The tumor growth curve. **C** Changes in tumor weight. **D** IHC was used to examine Ki67 expression (Scale bar: 100 μm). **E**, **F** RT-qPCR analysis of KDM4A-AS1 and AURKA expression. **G** Western blot analysis of AURKA, PI3K/AKT pathway proteins, and EMT-related proteins. Results expressed as mean ± SD for at least triplicate experiments. *P < 0.05, **P < 0.01, ***P < 0.001.

For exploring how KDM4A-AS1 contributes to HCC development in vivo, BALB/C male nude mice were injected subcutaneously with the stable HCC cells transfected with sh-E2F1, OE-KDM4A-AS1, and negative controls. As seen, compared with negative control group, repression of E2F1 inhibited volume and weight in vivo. However, these parameters were increased after overexpression of KDM4A-AS1 **(**Fig. 7A–C). IHC staining further showed that compared to sh-NC + OE-NC group, Ki67 expressed a weaken intensity in HCC tissues with E2F1 downregulation, indicating that E2F1 depletion could inhibit HCC cell proliferation, whereas this effect was abolished by KDM4A-AS1 overexpression (Fig. 7D). The levels of KDM4A-AS1 and AURKA in mice co-transfected with sh-E2F1 and OE-KDM4A-AS1 were greatly elevated compared with mice only treated with sh-E2F1 (Fig. 7E, F). Compared with negative control group, knockdown of E2F1 down-regulated AURKA, p-PI3K, and p-AKT protein levels in vivo, whereas overexpressed KDM4A-AS1 reversed these effects caused by E2F1 silencing **(**Fig. 7G**)**. In addition, E2F1 silencing induced a decrease in Vimentin expression and a increase in E-cadherin expression, which was abolished by KDM4A-AS1 upregulation (Fig. 7G). Thus, KDM4A-AS1 overexpression overturned the alleviated effects of E2F1 silencing on tumor formation in vivo through PI3K/AKT signaling pathway.

## Discussion

**Fig. 8:**
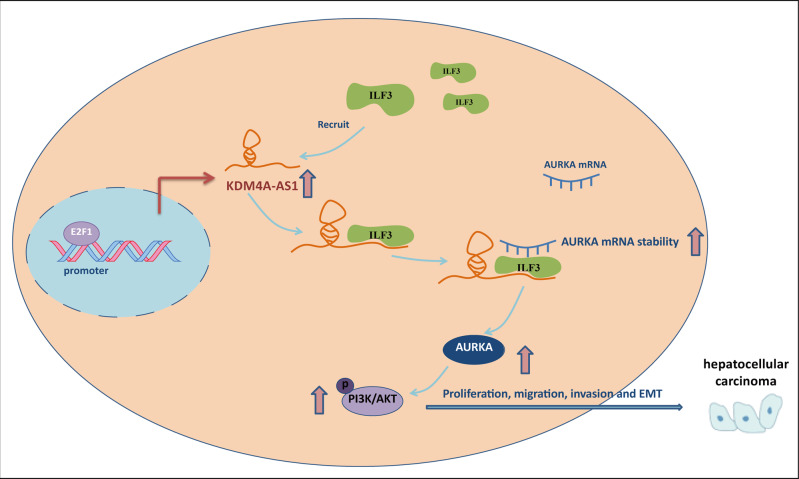
The mechanism diagram. E2F1 transcriptionally activated KDM4A-AS1. The up-regulated KDM4A-AS1 stabilized the AURKA mRNA by recruiting ILF3, and then promoted HCC cell proliferation, migration, invasion, and EMT via PI3K/AKT pathway.

HCC is the most common pathological type of primary liver cancer and one of the leading causes of cancer-related death [24]. In recent years, the incidence and mortality of HCC are generally increasing [25]. Early-stage HCC can be treated by surgery or ablation, but for advanced HCC, the available treatments are all palliative [26]. Therefore, identifying sensitive biomarkers in HCC is essential for its early detection and therapy. Evidence indicates that lncRNAs can be used as potential biomarkers for the prognosis and diagnosis of HCC patients [27]. Here, we discovered that KDM4A-AS1 was significantly increased in HCC tissues and cells. In addition, KDM4A-AS1 was transcriptionally activated by E2F1. Moreover, KDM4A-AS1 recruited ILF3 to stabilize AURKA mRNA and activated PI3K/AKT signaling pathway, inducing HCC cells to undergo EMT (Fig. 8).

Recent studies have found that aberrantly expressed lncRNAs were involved in the progression of HCC. LncRNAs can regulate the occurrence and development of HCC by recruiting RBPs. For instance, lncRNA DLEU2 promoted HCC progression through binding to EZH2 [28]. LncRNA SPRY4-IT1 induced HCC cell proliferation and metastasis via RNA-binding protein HNRNPL [29]. Here, KDM4A-AS1 is a relatively novel lncRNA and is considered an oncogene [30]. Recent data have proven that depletion of KDM4A-AS1 reduced cell growth and migration in castration-resistant prostate cancer [10]. As for HCC cells, KDM4A-AS1 was found to be associated with a poor prognosis of primary hepatic carcinoma [31]. Moreover, clinical studies have found that KDM4A-AS1 was significantly correlated with m6A modification in HCC [32]. It was also found that KDM4A-AS1 played a key role in promoting cell proliferation and invasion in vitro, as well as HCC growth and lung metastasis in vivo [23]. Similar to the previous findings, our data demonstrated that high expression of KDM4A-AS1 was positively correlated to poor prognosis in HCC. Moreover, KDM4A-AS1 downregulation greatly repressed cell growth and EMT in vitro, and tumor growth in vivo, indicating its involvement in HCC progression. These findings suggest that KDM4A-AS1 is a key candidate for the diagnosis and treatment of HCC. ILF3 is a double-stranded RNA-binding protein and is also associated with HCC development. A recent study identified that lncRNA ILF3-AS1 increased ILF3 mRNA stability and promoted HCC development [33]. We also demonstrated that KDM4A-AS1 is directly associated with ILF3 mRNA.

AURKA is a serine-threonine kinase that plays an essential role in maintaining chromosomal stability [34], as well as the occurrence and development of many malignancies, including HCC. For instance, AURKA promoted EMT and cancer stem cell behaviors via the PI3K/AKT pathway in HCC [35]. In our research, AURKA was verified as a target of ILF3 in HCC cells. Besides, KDM4A-AS1 elevated the stability of AURKA mRNA by recruiting ILF3 in HCC cells. In addition, we found that the suppression of HCC cell proliferation and metastasis mediated by ILF3 depletion was relieved by AURKA overexpression. Thus, we identified a KDM4A-AS1/ILF3/AURKA axis in HCC. What’s more, the PI3K/AKT signaling pathway is crucial for HCC progression [36]. AURKA could activate PI3K/AKT signaling pathway in various cancers. For example, AURKA mediated esophageal squamous cell carcinoma progression through PI3K/AKT [37]. Another study in bladder cancer demonstrated that active cathepsin B activated the AURKA/PI3K/AKT axis and promoted angiogenesis [38]. Similarly, our results indicated that the oncogenic role of KDM4A-AS1 in HCC was associated with AURKA/PI3K/AKT pathway.

E2F transcription factors play a key role in controlling many cellular functions associated with cell cycle progression [39]. As an important member of E2F family, E2F transcription factor 1 (E2F1) can regulate many cellular processes [40]. It has been reported that the transcription of lncRNAs could be regulated by E2F1 [41]. In our study, E2F1 was identified as an upstream regulator of KDM4A-AS1 and positively modulated its expression, leading to the PI3K/AKT pathway activation. As a transcription factor, E2F1 is essential for tumor growth and metastasis. In HCC, E2F1 plays a carcinogenic role [42]. E2F1 induced HCC proliferation by activating PKCα phosphorylation [43]. Moreover, E2F1 promoted HCC cell proliferation, migration, and invasion by activating the PI3K/AKT/mTOR signaling pathway [44]. Subsequently, we observed that E2F1 expression was increased in HCC cells. We further discovered that E2F1 promoted HCC cell EMT through transcriptional activation of KDM4A-AS1. Notably, KDM4A-AS1 stabilized ubiquitin carboxyl-terminal hydrolase 14/androgen receptor complex to promote tumor growth in castration-resistant prostate cancer [10]. Deubiquitination, as one of the important forms of protein post-translational modification, is the reverse process of ubiquitination, in which the substrate ubiquitin is removed by deubiquitinase [45]. The deubiquitination process was reported to be related to HCC cell proliferation, invasion, and metastasis [46]. There is evidence that E3 ubiquitin ligase CBLC positively regulated the stability of AURKA via ubiquitination in lung adenocarcinoma [47]. However, in addition to affecting the stability of AURKA mRNA, whether KDM4A-AS1 plays a role in promoting HCC by affecting the ubiquitination level of AURKA protein deserves further investigation.

To conclude, we identified that KDM4A-AS1 acted as a pivotal oncogene in HCC. E2F1 transactivated KDM4A-AS1, which contributed to the EMT of HCC cells by recruiting ILF3 and stabilizing AURKA mRNA. Additionally, KDM4A-AS1 promoted tumor formation by activating the PI3K/AKT pathway. Thus, KDM4A-AS1 may serve as a novel therapeutic target for HCC patients.

## Availability of data and materials

All data generated or analyzed during this study are included in this article. The datasets used and/or analyzed during the current study are available from the corresponding author on reasonable request.
